# Hypermobility of the first metatarsal bone in patients with Rheumatoid arthritis treated by lapidus procedure

**DOI:** 10.1186/1471-2474-13-148

**Published:** 2012-08-20

**Authors:** Stanislav Popelka, Rastislav Hromádka, Pavel Vavřík, Vladislav Barták, Stanislav Popelka, Antonín Sosna

**Affiliations:** 1 1st Orthopaedic Clinic, 1st Faculty of Medicine, Charles University in Prague, V Úvalu 84, Prague 5, 150 06, Czech Republic; 2 Institute of Anatomy, 1st Faculty of Medicine, Charles University in Prague, Prague, Czech Republic; 3 2nd Faculty of Medicine, Charles University in Prague, Prague, Czech Republic

**Keywords:** Lapidus procedure, Instability of the cuneometatarsal joint, Hypermobility of the first metatarsal bone, Rheumatoid arthritis, Hallux valgus

## Abstract

**Background:**

Foot deformities and related problems of the forefoot are very common in patients with rheumatoid arthritis. The laxity of the medial cuneometatarsal joint and its synovitis are important factors in the development of forefoot deformity. The impaired joint causes the first metatarsal bone to become unstable in the frontal and sagittal planes. In this retrospective study we evaluated data of patients with rheumatoid arthritis who underwent Lapidus procedure. We evaluated the role of the instability in a group of patients, focusing mainly on the clinical symptoms and X-ray signs of the instability.

**Methods:**

The study group included 125 patients with rheumatoid arthritis. The indications of the Lapidus procedure were a hallux valgus deformity greater than 15 degrees and varus deformity of the first metatarsal bone with the intermetatarsal angle greater than 15 degrees on anterio-posterior weight-bearing X-ray.

**Results:**

Data of 143 Lapidus procedures of 125 patients with rheumatoid arthritis, who underwent surgery between 2004 and 2010 was evaluated. Signs and symptoms of the first metatarsal bone instability was found in 92 feet (64.3%) in our group. The AOFAS score was 48.6 before and 87.6 six months after the foot reconstruction. Nonunion of the medial cuneometatarsal joint arthrodesis on X-rays occurred in seven feet (4.9%).

**Conclusion:**

The Lapidus procedure provides the possibility to correct the first metatarsal bone varus position and its instability, as well as providing the possibility to achieve a painless foot for walking. We recommend using the procedure as a preventive surgery in poorly symptomatic patients with rheumatoid arthritis in case of the first metatarsal bone hypermobility.

## Background

**Figure 1:**
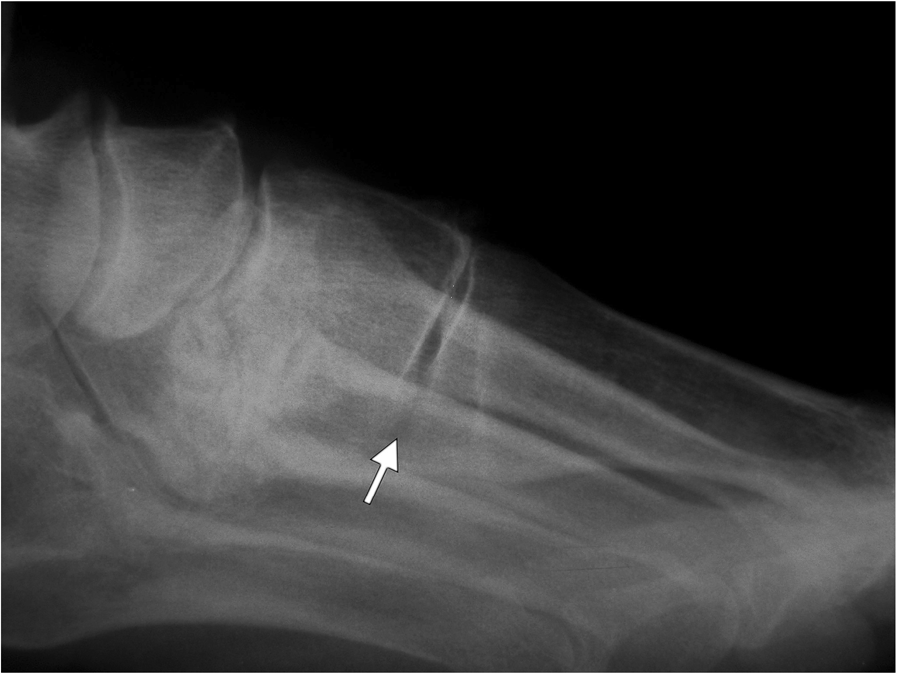
**Plantar gapping of medial cuneometatarsal joint.** Lateral X-ray weight bearing picture shows the plantar gapping (wedging) of the medial cuneometatarsal joint (white arrow).

Foot deformities and related problems of the forefoot are very common in patients with rheumatoid arthritis. Rheumatoid inflammation causes overall weakness of connective tissue and typical deformity of the foot. The laxity of the first cuneometatarsal (CM) joint and imbalance of muscles around the first ray contributes to the emergence of the hallux valgus. The impaired CM joint causes the first metatarsal bone (MTT) to become unstable in the frontal and sagittal planes [1-3]. The severe hypermobility of the first MTT bone can lead to luxation of the metatarsophalangeal joint (MTP) of the great toe (Figure 1).

The cuneometatarsal joint dysfunction and its role in development of the forefoot deformity is frequently discussed in the literature [2-7]. Hypermobility of the first MTT contributes significantly to the development of hallux valgus and plays an important role even in its treatment. Morton [8] in 1928 was the first to describe hypermobility and instability of the first MTT in the sagittal plane. Lapidus [9,10] found that increasing hypermobility of the first MTT leads to development of hallux valgus. Voellmicke [11] prefers the term “dorsal instability of the first MTT” over the term hypermobility, which is more appropriate for overall joint hypermobility.

However, the diagnosis and measuring hypermobility of the first MTT is very complicated. Various measurement aids have been proposed. Some are rather precise, while others are less precise and difficult to use in clinical practice [12-16].

In this retrospective study we evaluated data of patients with rheumatoid arthritis who underwent Lapidus procedure [9,10] due to progression of subjective symptoms. We evaluated the role of the instability in a group of patients focusing mainly on the clinical symptoms and X-ray signs of the first metatarsal bone instability.

## Methods

Our patient group included 125 patients treated by 143 Lapidus procedures. All patients were examined before the operation by authors. Only patients who met the objective and subjective criteria were included in the study. Objective inclusion criteria of the study and indications for the Lapidus procedure were the hallux valgus deformity greater than 15 degrees and varus deformity of the first MTT with the intermetatarsal angle greater than 15 degrees on anteroposterior weightbearing X-rays. Other inclusion criteria were rheumatoid arthritis and progression of the forefoot deformity prior to the operation.

Subjective indications for the surgery were progression of forefoot pain last three months and shortening of the maximum walking distance. All operations were done by the first two authors at the Orthopaedic Clinic, First Faculty of Medicine, Motol University Hospital in Prague, Czech Republic. The study was authorized by Ethics Committee on Research Project by Motol University Hospital, Reference number EK-190/12.

**Figure 2:**
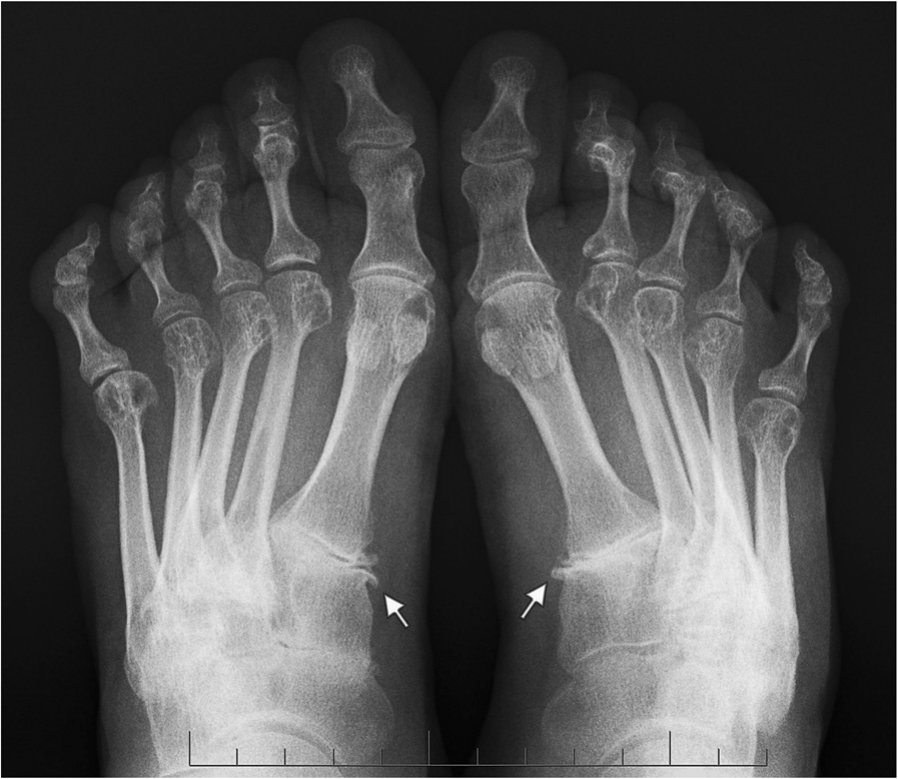
**Sign of the metatarsophalangeal instability.** Anteroposterior X-ray of a 40 year old woman with rheumatoid arthritis shows signs of the metatarsophalangeal instability, osteophytes of both medial cuneometatarsal joints (white arrows).

The primary focus of the preoperative clinical examination was the signs and symptoms of the medial CM joint instability. We noticed palpable cracking and snapping around the CM joint when vertical and horizontal pressure was applied. The preoperative X-ray examination included anterior-posterior and lateral projections of the weightbearing and nonweightbearing foot with measurements of the first intermetatarsal angle. The intermetatarsal angle increase was measured on AP views. The measurement of the angle was based on axes of the first and the second metatarsal bone. Each of lines was drawn connecting the center of the articular surface of the metatarsal head and the center of the proximal articulation as the longitudinal axis of the metatarsal [17,18]. Lateral views were focused on the asymmetry of the CM joint gap – plantar gapping [1] (Figure 1). The presence of osteophytes around the joint was also noted (Figure 2).

The operations were performed under general anesthesia, spinal anesthesia or foot block anesthesia [19] between 2004 and 2010. A longitudinal dorsomedial approach at the level of the CM joint was used to perform the procedure. The incision was elongated to the medial side of the first MTP joint. The tendon of the extensor hallucis longus muscle was pulled laterally with a retractor and the capsule of the CM joint was then released. Osteophytes on margins were removed to clarify orientations of joint surfaces and the bunion of the first metatarsal head was resected. Wedge resection on the proximal joint surface of the first MTT bone was then performed with respect to its latero-plantar orientation. The destroyed surface of the medial cuneiform bone was sliced off according to its original orientation and remains of the cartilage were removed.

An important part of the operation was the release of the lateral structures of the first MTP joint. The release was done according to the aforementioned medial approach or by an independent approach in the first interdigital web space. In the case of resection of the MTT heads, the lateral structures were released from the plantar approach. The release included; a lateral capsule incision with release of the lateral sesamoid ligament, partial tenotomy of the lateral conjoined tendon and release of the deep transverse intermetatarsal ligament. The appropriate level of the release was approximately five degrees of great toe varus in the MTP joint. The dorsal longitudinal interdigital approach was used to perform a Weil operation [20-22] when indicated or a transversal plantar elliptical approach at the level of MTT heads was used to remove the second to fifth metatarsal heads.

The resected CM joint was fixed by memory staples, two screws or two K-wires after reduction. Following the surgery a soft support bandage was applied. The Redon drain was removed the next day, thus allowing patients to walk with crutches without weightbearing on the operated foot. Full weightbearing of the foot was permitted approximately six weeks after the surgery or in case of healing signs on X-rays. Patients were examined at regular post surgical intervals at two weeks, six weeks, three months and six months. Evaluation of subjective symptoms and objective findings before and after the surgery were based on the American Orthopaedic Foot and Ankle Society (AOFAS) score [23] and X-rays. Measurements of the intermetatarsal angle on full weightbearing X-rays and outcome of the operations for the study were evaluated at six months follow up. The dependence of the intermetatarsal angle decrease and postoperative AOFAS score was evaluated with Student's *t*-test.

## Results

We evaluated data of 143 Lapidus procedures of 125 patients with rheumatoid arthritis, who underwent surgery between 2004 and 2010. The group included 120 women and five men. The right foot was operated on in 88 cases and left foot in 55 cases. In 18 patients the surgery was performed on both feet. The average patient age at the time of the surgery was 58.5 years (range, 38 to 71).

Signs and symptoms of first MTT instability in clinical and X-ray examinations were found in 92 feet (64.3%) in our group (Table 1).

**Table 1:** Lapidus procedure and hypermobility

	**Number of patients**	**Hypermobility**
Lapidus procedure and soft procedure of first MTP joint	69	31 (33.7 %)
Lapidus procedure and resection of MTT heads	45	39 (42.4 %)
Lapidus procedure and arthrodesis of the first MTP joint	14	13 (14.1 %)
Lapidus procedure and Weil osteotomies	11	9 (9.8 %)
Lapidus procedure and Keller's procedure	3	0 (0 %)
Lapidus procedure and Akin procedure	1	0 (0 %)
All procedures	143	92

Combination of the forefoot procedures with Lapidus procedure and their coincidence with the medial cuneometatarsal joint hypermobility.

MTP - metatarsophalangeal joint; MTT -metatarsal bone.

We used two Kirschner wires in eight cases and two screws in 15 cases for fixation of the arthrodesis. In 120 cases we used two memory staples introduced in two perpendicular planes (sagittal and transversal).

**Figure 3:**
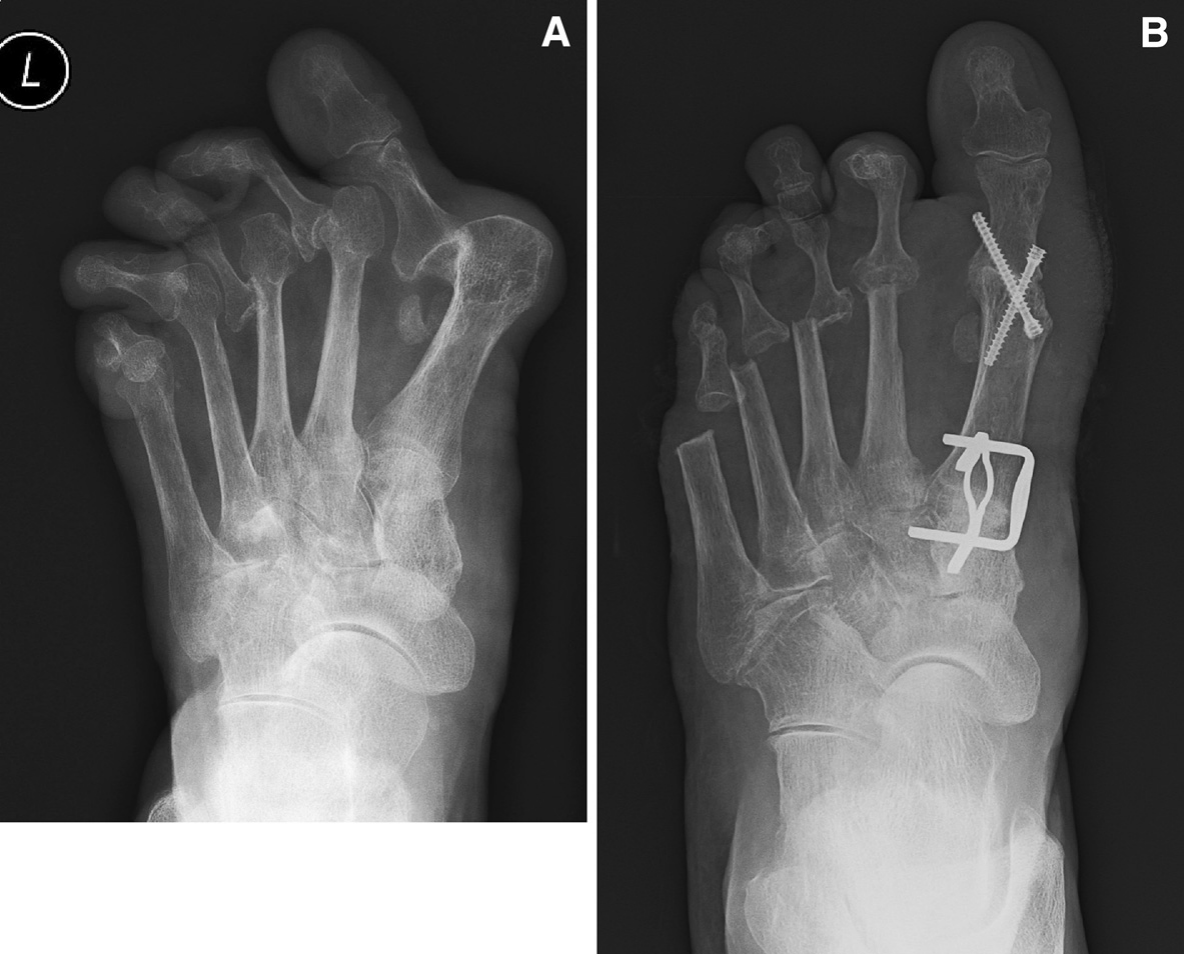
**A**. **Preoperative X-ray.** Preoperative anteroposterior X-ray of left foot shows severe deformity of the forefoot with dislocations of all metatarsophalangeal joints. **3B**. **Postoperative X-ray.** Postoperative anteroposterior X-ray of left foot shows arthrodesis of the medial cuneometatarsal joint fixed by two memory staples, arthrodesis of the first metatarsophalangeal joint fixed by two screws and resected second through the fifth metatarsal heads.

In 126 cases the operation was not combined with any bone procedure for the hallux valgus deformity, but only with the soft procedure of the lateral and medial structures of the first MTP joint. In 14 cases, due to severe valgus deformity of the great toe or severe subluxation, arthrodesis of the MTP joint was performed (Figure 3A, 3B). In four of those cases two K-wires were used, in one case we used memory staple and in ten cases two Barouk screws to fix the arthrodesis. In three cases we performed resection of the MTP joint [24,25] (Keller’s procedure) when patients with considerably restricted physical activity refused the arthrodesis of the first MTP joint. In one case the Akin procedure [26] of the great toe was done.

**Figure 4:**
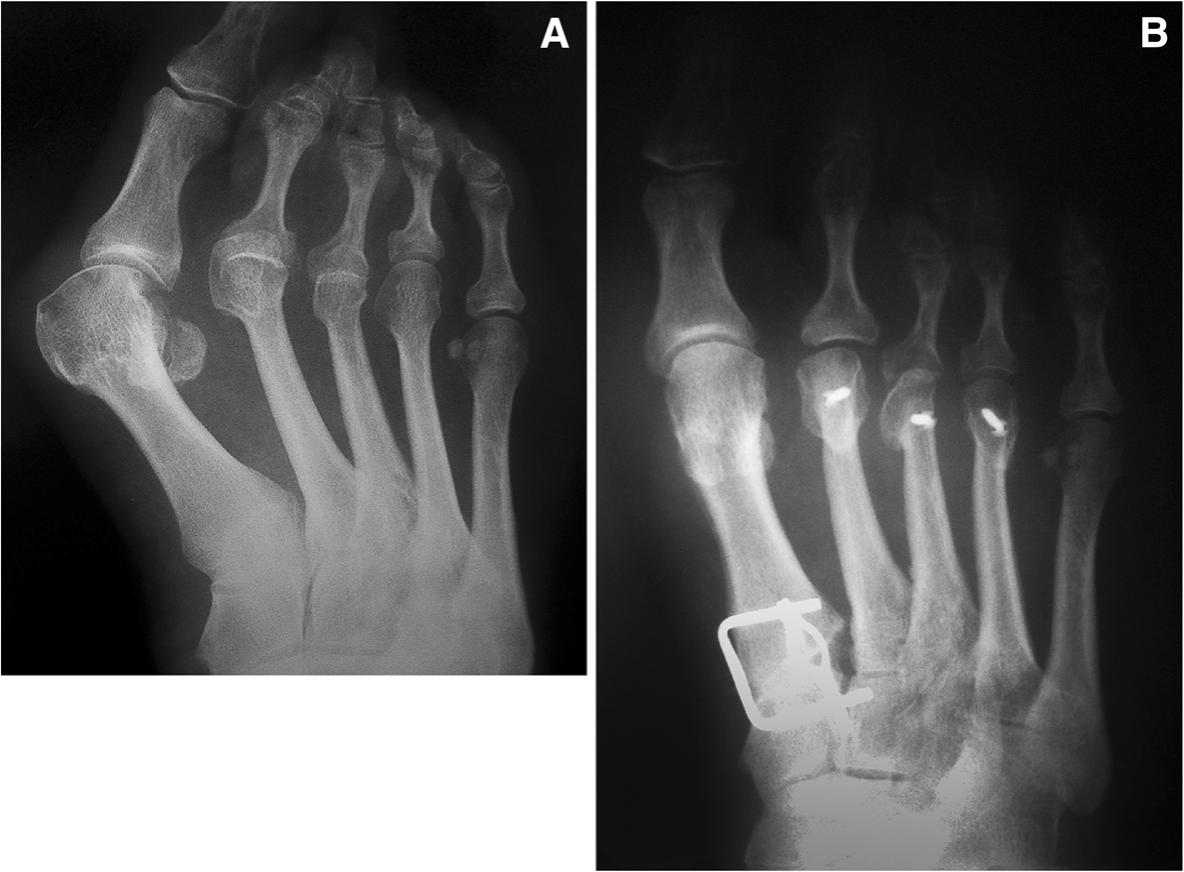
**A Preoperative X-ray.** Preoperative anteroposterior X-ray of right foot shows subluxation of the first metatarsophalangeal joint and dislocation at the second and third metatarsophalangeal joints. **B**. **Postoperative X-ray.** Postoperative anteroposterior X-ray of right foot shows the arthrodesis of the medial cuneometatarsal joint fixed by two memory staples and Weil osteotomy of the second to fourth metatarsal bones. Subluxation of the first metatarsophalangeal joint was resolved by the lateral release of the joint.

The Lapidus procedure was combined with several other procedures, which were performed on lesser toes as well as on the metatarsal bones. In 45 cases we combined the Lapidus procedure with the second through fifth MTT head resection using the transversal plantar approach. In 11 cases a Weil osteotomy [20-22] was done on the second through the fifth MTT bones to correct the length of each metatarsal, maintaining the Maestro line [20,27] (Figure 4A, 4B). In 25 cases of hammer toes the head of the phalanx was resected.

The intermetatarsal angle was evaluated during preoperative X-ray examination and six months after the procedure. The mean angle before the operation was 24 degrees (range, 15 to 35 degrees) and the mean six months after the surgery was 11 degrees (range, 8 to 14). In 10 cases the angle was not reduced to normal level owing to insufficient resection of the CM joint.

The mean of AOFAS score was 48.6 (range, 46.2 to 50.1) before the foot reconstruction and 87.6 (range, 85.2 to 91.2) six months after surgical procedures. Statistically significant dependence between postoperative AOFAS score and the decrease of intermetatarsal angle was not found.

Complications included one deep infection, which required surgical treatment and removal of osteosynthetic material. Delayed healing of the wound over 21 days was recorded in 15 cases (10.5 %). Nonunion of the arthrodesis on X-rays occurred in seven feet (4.9 %) at the 6 months follow up. In these cases two cases screws, one case Kirschner wire and in four cases memory staples for fixation were used.

## Discussion

A foot is often the first part of the body affected in patients with rheumatoid arthritis. Pain and deformity of the foot are early symptoms of the disease in many cases. Progression of the forefoot deformity usually progresses to medial cuneometatarsal joint instability.

The hypermobility of the first MTT is the subject of frequent discussion [16,28-30]. In patients with rheumatoid arthritis, synovitis of the first MTP and CM joints together with a foot muscle imbalance leads to first MTT instability. The issue is whether the instability of the CM joint is the cause of the hallux valgus or its consequence.

Confirming the diagnosis of instability in clinical practice can be difficult, because the surgeon has to frequently rely on the physical examination and weight-bearing X-ray of the foot. Various hypermobility measurement aids and devices have been proposed, but they are difficult to use in clinical practice.

Klaue [13] demonstrated a relationship in which increased mobility of the first MTT increases the hallux valgus deformity. He designed a device to measure first MTT mobility as well. Physiological dorsal excursion of the first MTT is up to 8 mm and a greater range of motion is considered pathological and indicates hypermobility.

The important stabilizing element is the plantar fascia, which becomes loose as the valgus deformity of the great toe progresses. According to Sarrafian [31] the plantar aponeurosis is one of the stabilizers of the forefoot and the great toe position. This condition was also confirmed by Grebing [32] who studied the impact of the plantar aponeurosis on stability of the first MTT. Using a Klaue device, they compared a group of patients on which plantar fasciectomy had been performed due to plantar fibromatosis to a group of patients on which no such surgical procedure had been performed. Grebing found that the plantar aponeurosis plays an important role in ensuring the stability of the CM joint. Hypermobility of the first MTT was present more often in the case of patients who had undergone the surgery than those who had not.

When examining the instability of the first MTT, the position of the talus is important. Grebing [32] report in their work that hypermobility of the first MTT changes with the position of the talus during examination. Mobility of the first MTT is lower in dorsiflexion and it is greatest in plantar flexion. Their conclusions are important during the foot examination when the ankle has to be kept in a neutral position.

First MTT instability was diagnosed in 92 feet (64.3%) in our group of patients. Patients with rheumatoid arthritis can have progression of forefoot deformity and pain even without instability of the CM joint. Lapidus operation can be important for stability of the whole forefoot in these cases. During the procedure not only is the instability resolved, but the position of the MTT bone as well. The operation is based on arthodesis of the CM joint with bone resection of the joint surfaces and internal fixation, but it can be complicated to achieve correct postoperative orientation of the first MTT bone. Appropriate bone resection during the procedure is essential for a positive postoperative outcome of the surgery. We always remove wedged bone block from the proximal part of the first MTT during the surface resection. The wedge is orientated laterally to adjust the varus position of the metatarsal bone. The high of the block is directly proportional to valgosity of the great toe and inclination is directly proportional to varosity of the first metatarsal bone. Insufficient resection can lead to elevation of the first MTT in the sagittal plane. The removal of all osteophytes and in some cases even the medial part of the middle cuneiform bone is important for the reduction of the gap after resections. In some cases, resection of the lateral part of the first MTT base is also required. The first intermetatarsal angle has to be corrected unless the relapse of the hallux valgus is significant.

For the purpose of this study we used two Kirschner wires, two screws or two staples for the internal fixation, but standardly use only two memory staples introduced in the sagittal and horizontal planes. Each staple has to insert through both the cortical bones in the MTT and the CM bones. The fixation using two screws was sometimes problematic, particularly in the case of rheumatoid patients where osteoporosis is present. If the memory staples are inserted only through one cortical bone, the fixation is not firm enough. The staple cannot ensure firm fixation, which can be crucial in case of a patient with rheumatoid arthritis.

In general, various osteosynthetic materials are used for arthrodesis fixation (Kirschner wires, screws, various plates, staples) or external fixation is used [29,33,34]. Fixation using two memory staples has proven the most successful. The memory staple fixating is quick, simple and, according to our experience, it is also reliable.

Outcomes of the Lapidus procedure cannot be evaluated with real precision, especially in the case of patients with rheumatoid arthritis, due to comprehensive affliction to the foot. We did not find any significant statistical dependence between the decrease of intermetatarsal angle and postoperative AOFAS score in our study.

Shi et al. [35] assessed the outcomes of the procedure in 21 patients with rheumatoid arthritis. They report pain relief and the possibility to wear normal shoes. They also evaluated changes identified by the X-ray examination – the angle of valgus deformity of the great toe, the intermetatarsal angle between the first and second MTT (reduction from 13 to 8.3 degrees) and the angle between the first and fifth MTT bones (reduction from 32.2 to 21.1 degrees). They report that 17 patients reported significant pain relief and 16 patients are able to wear normal shoes.

Myerson and Badekas [36] discuss hypermobility of the first MTT as a predisposing factor for the emergence of hallux valgus, in particular in combination with affliction of the ligaments and muscle imbalance. Hypermobility frequently occurs in adolescents with hallux valgus, in particular when there is an increased intermetatarsal angle between the first and second metatarsals. The authors describe the clinical and X-ray indicators of the hypermobility and the use of Lapidus procedure to treat patients.

A discussed issue is the occurrence of nonunion after performing the Lapidus procedure – the occurance of nonunion ranged between 3.3% and 9%. Patel [37] reviewed a set of 227 surgical procedures where screws were used for arthrodesis fixation. In 12 cases (5.3 %) nonunion developed.

In our group of patients with rheumatoid arthritis we recorded nonunion in seven cases (4.9%) and persistent swelling of the dorsum of the foot occurred in 12 (8.4%) operated feet.

## Conclusions

Deformities of the great toe and forefoot are very frequent in patients with rheumatoid arthritis. Typically, patients suffer from a polyarticular affliction. The objective of foot surgery is to: achieve a plantigrade foot position, allow the patient to wear normal or individual shoes, achieve painless walking and standing and also to prevent asymmetric overloading of the adjacent joints. The Lapidus procedure allows correction of the varus deformity of the first MTT thus correcting the valgus position of the great toe and ensuring sufficient stability of the first ray. We recommend using the procedure as a preventive surgery in poorly symptomatic patients with rheumatoid arthritis in case of the first metatarsal bone hypermobility.

## Abbreviations

CM: Cuneometatarsal; MTT: Metatarsal bone; MTP: Metatarsophalangeal joint; AOFAS: American Orthopaedic Foot and Ankle Society.

## Competing interest

The authors declare that they have no financial and non-financial competing conflict of interest. The study was supported by the project of Ministry of Health, Czech Republic, for conceptual development of research organization 00064203 (University Hospital Motol, Prague, Czech Republic).

## Authors’ contributions

SP, RH – surgeons, who performed Lapidus operations, PV, VB, SP Jr, AS -investigators to evaluate data of procedures. VB - responsible for statistics. All authors contributed to the writing of the manuscript. All authors read and approved the final version of the manuscript.

## Pre-publication history

The pre-publication history for this paper can be accessed here:

http://www.biomedcentral.com/1471-2474/13/148/prepub
